# Design and Implementation of Chinese Common Braille Translation System Integrating Braille Word Segmentation and Concatenation Rules

**DOI:** 10.1155/2022/8934241

**Published:** 2022-05-17

**Authors:** Ju-Xiao Zhang, Hai-Feng Chen, Bing Chen, Bei-Qin Chen, Jing-Hua Zhong, Xiao-Qin Zeng

**Affiliations:** ^1^College of Information & Mathematics Science, Nanjing Normal University of Special Education, Nanjing 210038, Jiangsu, China; ^2^Nanjing Dian-Ming Software Technology Co, Ltd, Nanjing 210038, Jiangsu, China; ^3^China Braille & Sign Language Research & Application Center, Nanjing Normal University of Special Education, Nanjing 210038, Jiangsu, China; ^4^College of Special Education, Beijing Union University, Beijing 100075, China; ^5^College of Computer and Information, Hohai University, Nanjing 210038, Jiangsu, China

## Abstract

An important sign of the accessibility of Braille information is the realization of the mutual translation between Chinese and the Braille. Due to the irregularity and uncertainty of the Prevailing Mandarin Braille, coupled with the lack of a large-scale Braille corpus, the quality of Chinese-Braille translation seems to be poor. In July 2018, the National Language Commission released the “Chinese Common Braille Scheme” and advocated replacing the “Prevailing Mandarin Braille.” Aimed at improving translation accuracy, this research, which is based on the self-built Chinese Common Braille corpus and combined with the HanLP (Han Language Processing) dictionary and the Chinese-Braille word corpus (a Braille word segmentation and concatenation dictionary for generating a unigram language model), uses the n-gram language model to design and implement a Chinese-Braille intertranslation system that integrates Chinese and Braille Word Segmentation and Concatenation Rules. More importantly, this research proposes an experimental plan for improving the Braille Word Segmentation and Concatenation Rules using a Chinese-Braille word corpus. Experiments show that in the field of educational literature, the accuracy rate of translation from Chinese to Chinese Common Braille has reached 95.01%, and the accuracy of Chinese Common Braille to Chinese translation has reached 90.15%.

## 1. Introduction

An important sign of the accessibility of Braille information is the mutual translation between Chinese and Braille so that no significant differences can be found between the original and translated Chinese characters on smart devices. Braille is a special script with the properties of the host. Braille generally does not exist independently (there is no Braille used in a country that can be separated from a certain language, and there is no Braille that is used internationally across languages), and there are both associations and differences with the host language. The appearance of Braille is the same all over the world, but the difference in the host language makes the Braille of the corresponding language completely different from others. The informatization of English Braille is easy to complete, and the level of informatization is also high, so that blind people who use English can be well educated, which helps to promote social equity and to achieve great social significance.

At present, the Braille that bonds with the Chinese is collectively referred to as “Chinese-Braille,” and there are three main types, Prevailing Mandarin Braille, the double spelling Braille, and the Chinese Common Braille Scheme in 2018. The use of double spelling Braille is less often, and now the Prevailing Mandarin Braille is mainly used, and the use of the Chinese Common Braille is gradually promoted [1]. The Prevailing Mandarin Braille at most uses the three-cells Braille (initial, final, and tone, respectively) to represent a Chinese character and suffers from the following problems:

1. The general principle of tone is “generally not to mark the tone, but only marked when necessary” [2], which makes Braille expression rely on expert experience. Besides, there are principles but no norms. In particular, the understanding of the homophones in Chinese itself, no matter with marked tones or not, has to rely on “guessing” and therefore the ambiguity is increased.
2. Word segmentation and concatenation rules are not yet perfect. Unlike “characters” and “characters” that are not separated in Chinese, Braille draws on English word segmentation rules (Braille is similar to Pinyin in essence) by adding “blank cells” (or spaces) between words to reduce ambiguity. Braille word segmentation is not only based on semantics but also considers the tactile problem of “touching and reading” for blind people (reducing blank cells and improving reading speed). Therefore, it is necessary to concatenate words that are originally semantically separated, which is called Braille Word Segmentation and Concatenation Rules. For example, “引/无数/英雄/竞/折腰.” There are about 100 rules in the Braille Word Segmentation and Concatenation Rules, which are still not perfect, and they are often done manually by Braille experts.

The above-mentioned irregularities and uncertainties hinder the translation of Chinese to Braille and make it difficult to improve the Braille informatization at a certain level. Researchers have been looking for breakthroughs for many years, but the results are not obvious. This is also the original intention of the nation to promulgate and promote the Chinese Common Braille.

The Chinese Common Braille inherits the Prevailing Mandarin Braille as a whole and “upgrades” the Prevailing Mandarin Braille to some extent. Mainly, all characters are marked with tones, and the tones are written according to the initials. This not only reduces the randomness of Pinyin tone but also reduces the number of cells in Braille [1]. For example, the Pinyin of “更加” is “gèngjiā” while the Prevailing Mandarin Braille is 
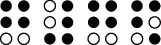
 without marking the tones for both characters. However, the characters with marked tones should be 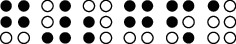. The Chinese Common Braille rule is that when the initial consonant is “*g*,” the tone is omitted so that the Common Braille should be 
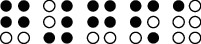
.

### 1.1. Research Background

Some scholars have carried out research on the basis of Prevailing Mandarin Braille, such as Zhou et al. [3] and Zhu et al. [4–6], starting from the rules, segmenting words according to Chinese semantics, and then converting Chinese words into Braille words. The accuracy from Chinese to Braille is high, but the accuracy from Braille to Chinese is not satisfactory. To solve the problem of word segmentation and concatenation, some scholars built a small-scale Braille word segmentation database and use the Trie tree to process word segmentation and concatenation [7]. Some are first-word frequency and word grading weighted word segmentation and then the combination of rules and statistics [8]. Some followed the Chinese-Pinyin-Braille conversion manner [9]. Some scholars used the Markov model to identify Chinese characters, and then the reverse maximum matching word segmentation method is used to segment Chinese words; scholars such as Wang Xiangdong combined the Chinese-Braille word segmentation, Braille Word Segmentation, and Concatenation Rules and tone information for higher translation accuracy [10]. Furthermore, there are also methods based on statistical machine learning to realize Chinese-Braille machine translation [11]. Another work proposes an algorithm that integrates Chinese word segmentation and Braille word segmentation [12, 13] to improve the accuracy. A deep learning-based technique that trains a bidirectional LSTM model achieved a word segmentation accuracy rate of 94.42% [14]. However, the Prevailing Mandarin Braille corpus is self-made, and no in-depth research has been carried out on the Chinese Common Braille. The China Disabled Persons' Federation project “Development of Common Braille Automatic Translation Software” hosted by Professor Xiao Hang developed a Common Braille automatic translation software by adopting a language model combining N-grams and maximum entropy, and good translation results have been achieved [15].

### 1.2. Outlines and Contributions

The remainder of this paper is arranged as follows. In Section 2, we will introduce the detailed information of the Chinese-Braille word corpus provided by the team of Professor Jing-Hua Zhong of Beijing Union University as we carried out our research based on this dataset. We will present the detailed procedures of our developed system in Section 3, where we will firstly introduce the details of translation from Chinese to Braille and vice versa. We then proposed our method for Braille word segmentation and concatenation. Specifically, we firstly deploy the n-gram model to segment Chinese words and then train a Braille word segmentation concatenation dictionary to generate a unigram language model for Braille word segmentation adjustment. By doing so, we aimed at improving the translation accuracy from Chinese to Braille. Finally, we improved the Braille Word Segmentation and Concatenation Rules by experiment. The details of the experiment are presented in Section 5. In terms of translation from Chinese Common Braille in the field of educational literature, the accuracy has reached 95.01% while the translation accuracy reached 90.15% when translating Chinese Common Braille to Chinese.

## 2. Material

### 2.1. Chinese-Braille Word Corpus

The team of Professor Jing-Hua Zhong of Beijing Union University has undertaken the National Social Science Fund major project “Research on the Construction of Chinese-Braille Corpus.” With the authorization of Professor Jing-Hua Zhong, this study uses the Chinese-Braille word corpus provided by him. We automatically extract words from literature, science, and other books to make a Chinese-Braille word corpus the word corpus and then manually reviewed and revised them. The corpus contains the information shown in Table 1.

This corpus is a Chinese-Braille word dictionary. The corpus contains the words extracted from the corpus text, and a mapping relationship is established.

### 2.2. Braille Word Segmentation and Concatenation Rules

The word segmentation and concatenation rules can be seen in Table 1 which lists the refinement and annotation of existing word segmentation and concatenation rules. The annotations are slightly different from the Chinese corpus, as shown in Table 2, which was made by Jing-Hua Zhong's team.

### 2.3. Construction of Braille Word Segmentation and Concatenation Dictionary

According to the Chinese-Braille word corpus, a Braille word segmentation and concatenation dictionary is established. In particular, the existing corpus is only a word corpus, and only a Braille unigram language model dictionary is constructed here. In this dictionary, the core is to count the frequency of words.

The number of word frequencies in Table 2 equals the sum of the number of frequencies of this type of word segmentation. “-” means unsubdivided statistics. More specifically, the numbers and punctuation marks in the Chinese-Braille word corpus are removed, only Chinese words are retained, the word frequency of each Braille word is counted, and it is stored as a Braille unigram language model dictionary, as shown in Table 3.

There are a total of 229,551 words in the corpus, 31,708 of which are the Chinese Common Braille Pinyin, 1,477 of which correspond to two or more different Chinese words, forming a total of 3,491 Chinese words, 1,477 of which have the same Pinyin and a total of 46,235 Chinese Common Braille words. The sum of the highest frequency of each Pinyin word is 46,235.

Table 4 shows the fragment content of the unigram Braille word segmentation and concatenation dictionary. The first column is the Chinese Common Braille Pinyin, the second column is the Chinese words connected according to the Braille word segmentation, the third column is the word segmentation type, the fourth column is the Braille ASCII Pinyin, and the last column is the word frequency.

## 3. Design of Chinese-Braille Translation System

The release of the Chinese Common Braille has fundamentally solved the problem of Braille marking. Therefore, in the translation process from Chinese to Braille, the core difficulty is the Braille Word Segmentation and Concatenation Rules [12].

The main methods of translation from Chinese to Braille are as follows:

1. Formally describe the concatenation rules of word segmentation [5]. The emphasis is on the formal description of word segmentation and concatenation rules and their application to word segmentation in Braille Pinyin. However, the word segmentation rules are not perfect, and new rules are still generating, making it more difficult for this method to improve the accuracy of word segmentation.
2. Extract word segmentation and concatenation from Braille corpus, use the word segmentation library to segment Chinese words, then implement pinyinization after word segmentation, and then convert to Braille. This method relies on an accurate and large-scale Braille corpus, and the Chinese Common Braille corpus is still continuous improvement [9].
3. Directly train word segmentation and concatenation rules from Chinese and Braille's dictionary (unlabelled corpora) through machine learning methods [11]. The Chinese word segmentation is combined with the Braille word segmentation, and the Braille Word Segmentation and Concatenation Rules adjusts the Chinese word segmentation to obtain the final Braille word segmentation, and the final translation accuracy is improved by 3.56% [12]. Satisfactory results were achieved without using the Chinese Common Braille corpus.

Based on the Chinese-Braille word corpus, combined with Method 2 and Method 3, the mutual translation between Chinese and Chinese Common Braille can achieve better results. Firstly, Chinese sentences are organized according to the Braille Word Segmentation and Concatenation Rules; secondly, Chinese words in Chinese Pinyin are marked (especially the words that are changed in the sentence must be in the same tone); finally, according to the Chinese Common Braille consonant representation rules, the Pinyin syllables are converted into Braille to achieve the “Braille” of Chinese Pinyin and other characters.

In the translation process, there are also some details that need to be coped with. For example, according to the representation rules of the Chinese Common Braille, the comparison table between Chinese Pinyin and the Chinese Common Braille, numerous tables have to be made, such as the comparison table between the numerical symbols and the Chinese Common Braille, the comparison table between the English letters and the Chinese Common Braille, and the comparison table between various symbols and the Chinese Common Braille. When brailing various characters, firstly, it is necessary to solve the problem of adding prompt symbols when Chinese, English, various numbers, symbols, and other text symbols are mixed; secondly, it is necessary to solve various noncontent symbols (such as hyphens), format prompts, etc.

### 3.1. Chinese-Braille Translation

The Chinese-Braille translation is just the direct translation from Chinese characters into Braille. The sentence can contain non-Chinese characters such as numbers, English, and punctuation marks, but there is a one-to-one correspondence between such information and Braille, where direct conversion can be applied. The accuracy, however, is very high already and will not be discussed here.

The biggest technical problem in Chinese-Braille translation is the Braille Word Segmentation and Concatenation Rules. With the simultaneous development of machine translation technology, Chinese word segmentation technology methods are divided into rule-based, statistical-based, and deep learning [16]. The rule-based method mainly depends on the dictionary, and the more classic ones are the maximum matching. The advantage is that it is simple, easy, and efficient; the disadvantage is that it depends on the accuracy and scale of the dictionary, and the recognition ability of ambiguous words is poor. The statistical methods rely on the corpus and use the corpus context information, word frequency, information entropy, etc., to perform word segmentation. The more classic ones are based on n-gram, maximum entropy model, hidden Markov model (HMMs) [17], conditional random field model (CRF), and so on. The advantage is that the accuracy rate is significantly improved, and the disadvantage is that it relies on a relatively large-scale corpus, which causes bias problems (maximum entropy model, hidden Markov model) or model complexity and low efficiency (CRF). The N-shortest tokenizer has a better effect than the shortest tokenizer and has a stronger ability to recognize named entities, but the speed is much slower; the CRF (conditional random field) methods usually have an accuracy and recall rate that are higher than 96% and have good new word recognition ability. In recent years, neural networks and deep learning methods are very eye-catching techniques [18]. Deep learning has made outstanding achievements in natural language processing, image recognition (especially medical image recognition [19, 20]), target detection, and so on. Deep learning includes Convolutional Neural Networks (CNNs) to Recurrent Neural Networks (RNNs) to long- and short-term memory neural network model LSTM [21] and improved variations of LSTM. The advantage is that the effect is good, and the disadvantage is that it relies on a large-scale corpus, long training time, and poor interpretability. Of course, there is also a combination of the above-mentioned methods to achieve good results and high efficiency as much as possible.

At present, there is only a Chinese-Braille vocabulary corpus, and there is a lack of a large number of well-known Braille corpora that have been correctly segmented and marked. We, therefore, proposed making full use of the existing Chinese dictionary and word segmentation system, which is conducive to the accuracy of Chinese-Braille translation. Based on the Chinese-Braille vocabulary corpus + HanLP dictionary, this research will use the n-gram language model to segment Chinese sentences and then use the Braille word segmentation normative dictionary to improve the accuracy of word segmentation, thereby improving the accuracy of Chinese-Braille translation.

**Figure 1:**
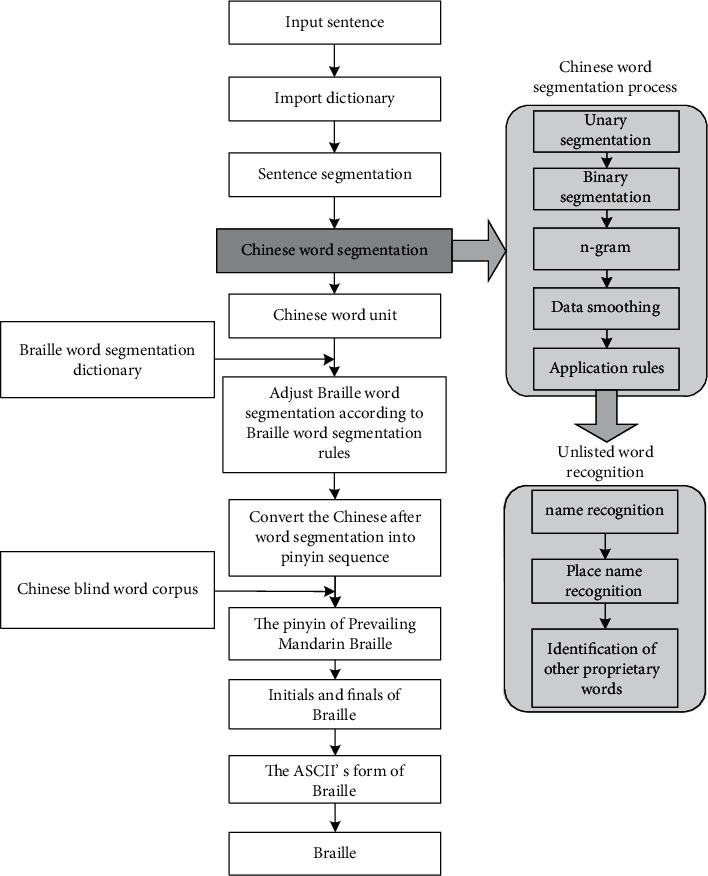
The translation process of Chinese-Chinese Common Braille.

As shown in Figure 1, the process of Chinese to Chinese Common Braille translation based on the Chinese-Braille word corpus is given. Starting from a Chinese sentence, we firstly use n-gram for training word segmentation (combining HanLP dictionary with Chinese-Braille vocabulary corpus) and then convert Chinese into Pinyin strings; then based on the Chinese-Braille vocabulary corpus, it is converted into a Chinese Common Braille Pinyin string, which fuses word segmentation and concatenation rules in Chinese-Braille word corpus. Finally, the Braille Pinyin string obtained after word segmentation is converted into ASCII Pinyin symbols and Braille symbols.

#### 3.1.1. Chinese Word Segmentation Using n-Gram Language Model [22, 23]

Suppose that the sentence *S*=*c*_1_*c*_2_ … *c*_*N*_ needs to be divided into Chinese words, and *c*_*i*_(1 ≤ *i* ≤ *N*) is a single Chinese character. The result after Chinese word segmentation *R*=*w*_1_*w*_2_ … *w*_*M*_,  (1 ≤ *M* ≤ *N*).

Assuming that during Chinese word segmentation, the probability of occurrence of the *w*_*j*_(1 ≤ *j* ≤ *M*) is related to all the previous words, that is, related to the previous *j* − 1 words [24], then the j-gram language model can be expressed as

(1) $$\begin{array}{c} P\left(w_{j}|w_{1}^{j-1}\right)\left(j>1\right). \end{array}$$

It can be known from the Bayesian formula that

(2) $$\begin{array}{c} P\left(w_{j}|w_{1}^{j-1}\right)=\frac{P\left(w_{j}\right)}{P\left(w_{1}^{j-1}\right)}. \end{array}$$

The probability of word *w*_*j*_ can be calculated from

(3) $$\begin{array}{c} P\left(w_{j}|w_{1},w_{2},\ldots ,w_{j-1}\right)=\;P\left(w_{j}|w_{j_{0}+1},w_{j_{0}+2},\ldots ,w_{j-1}\right), \end{array}$$

where *j*_0_=max(*j* − *n*, 0).

Given a sentence consisting of a sequence of *m* words *R*=(*w*_1_, *w*_2_,…, *w*_*M*_),  (1 ≤ *M* ≤ *N*) that can be denoted as *w*_1_*w*_2_ … *w*_*M*_, and its probability is (4):

(4) $$\begin{array}{c} P\left(R\right)=P\left(w_{1}\right)\prod_{2}^{M}P\left(w_{j}|w_{j_{0}+1},w_{j_{0}+2},\ldots ,w_{j-1}\right). \end{array}$$

It can be seen from the formula that the probability of forming a sentence is the product of the word probabilities of Chinese word segmentation. Theoretically, the larger the value of *n*, the better the segmentation effect, but the larger the value of *n*, the greater the amount of calculation, so that it cannot be calculated; in addition, the sparsity of the data is serious.

The magnitude of the model parameters is an exponential function (*N*^*n*^)  of the model length *n*, so *n* cannot be very large. For a word corpus with sufficiently large sample size, the probability can be calculated using the word frequency approximation:

(5) $$\begin{array}{c} P\left(w_{j}|w_{1}^{j-1}\right)=\frac{P\left(w_{j}\right)}{P\left(w_{j-n+1}^{j-1}\right)}\approx \frac{\text{count}\left(w_{j}\right)}{\text{count}\left(w_{j-n+1}^{j-1}\right)}, \end{array}$$

where count(*w*_*j*_) and count(*w*_1_^*j*−1^), respectively, represent the number of occurrences of *w*_*j*_  and *w*_1_^*j*−1^  in the corpus.

According to Markov's hypothesis, the possibility of several words forming a word only depends on one or a limited number of words that appear before it and has nothing to do with the following words. The current value of *n* is generally 2 or 3.

If it only depends on a word that appears before it, it is called a 2-gram. And if it only depends on the two words that appear before it, it is called a 3-gram.

When *n* = 2, (5) becomes

(6) $$\begin{array}{c} P\left(w_{j}|w_{1}^{j-1}\right)\approx \frac{\text{count}\left(w_{j-1},w_{j}\right)}{\text{count}\left(w_{j-1}\right)}. \end{array}$$

#### 3.1.2. Data Smoothing Algorithms [25]

There are two serious problems in the approximate calculation of formula (6): the first one is that the probability of words that do not appear is approximated to 0 (data sparsity); the second one is when count(*w*_*j*−1_, *w*_*j*_)=count(*w*_1_^*j*−1^), then *P*(*w*_*j*_*|w*_1_^*j*−1^)=1.

Therefore, the data needs to be smoothed. Data smoothing is to appropriately reduce the probability of each word in the sample and adjust the reduced probability value to the words that do not appear so that the probability of all words is not 0 and the sum of the probabilities is still equal to 1. Commonly used data smoothing techniques are Add-delta smoothing, Good-Turing smoothing, combined estimation, simple linear interpolation, Jelinek-Mercer smoothing, fallback model and Katz smoothing, etc.

This study employs Good-Turing smoothing, which is suitable for large vocabularies to generate multimodal distributions of observations.

*N* is the size of the original training sample data, and *n*_*x*_ is the number of words that appear *x* times in the training sample. Then,

(7) $$\begin{array}{c} N=\sum_{x=1}^{\infty }n_{x}x,\\ N=\sum_{x=0}^{\infty }n_{x}x^{*}=\sum_{x=0}^{\infty }\left(x+1\right)n_{x+1},\\ x^{*}=\left(x+1\right)\frac{n_{x+1}}{n_{x}}. \end{array}$$

Then, the probability of the word appearing *x* times in the sample is

(8) $$\begin{array}{c} P_{x}=\frac{x^{*}}{N}. \end{array}$$

Finally, the probability normalization of all words is processed:

(9) $$\begin{array}{c} \hat{P_{x}}=\frac{P_{x}}{\sum P_{x}}. \end{array}$$

#### 3.1.3. Chinese Word Segmentation Based on the 2-Gram Model of Word Segmentation and Concatenation Rules

**Figure 2:**
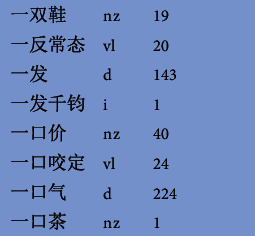
Fragments of unigram model dictionary.

**Figure 3:**
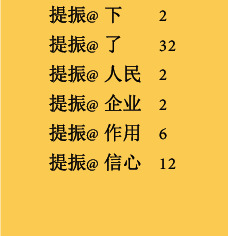
Fragments of bigram model dictionary.

As shown in Figure 1, assuming a Chinese sentence *C*=*c*_1_*c*_2_,…, *c*_*n*_，C is a sentence, and *c*_*i*_  is a character, based on the Chinese-Braille vocabulary corpus + HanLP dictionary, the specific process is as follows:

1. Divide characters: enter Chinese sentences and divide all words of the sentence into independent words. Spaces can be added after each word to distinguish Chinese, English, and punctuation marks.
2. Unary segmentation: use the unary language model dictionary (CoreNatureDictionary) in the dictionary and the maximum matching algorithm to match the characters with the dictionary words, and form a unary word network containing information such as part of speech, word frequency, etc. Combine English characters and numeric characters into atomic words, and words are represented by *w*, that is, *w*_*i*_=*c*_*j*_ … *c*_*k*_, (*j*, *k* ≥ 1).
3. The fragment content of the HanLP unary language model dictionary is shown in Figure 2.
4. The first column is the word, the second column is the type of the word, and the third column is the word frequency; if there is a second type of the word, the fourth column is the second type, the fifth column is the word frequency of this type, and so on.
5. Binary segmentation: according to the binary dictionary (CoreNatureDictionary.ngram), we continue to use the maximum matching algorithm to form a word graph (Figure 3), use @ to separate two words, and the probability of appearing as a common word, such as the word after “提振(boost)” is “信心(confidence)” which appears 12 times.
6. Chinese word segmentation of the 2-gram model.
7. Use the two dictionaries in HanLP (CoreNatureDictionary and CoreNatureDictionary.ngram). The maximum forward and backward algorithms can be used to segment the sentence to obtain two strings s1 and s2, respectively; if two different word sequences are obtained, the one with the highest probability is selected according to the bigram, which can eliminate part of the ambiguity.
8. Apply the above results and apply the rules to identify the spatial nouns.
9. Based on a name recognition dictionary, place name dictionary, and proper noun dictionary, use a two-layer HMM (Hidden Markov Model). Taking the word sequence as the observation sequence and the dictionary word probability sequence as the hidden sequence, when the model predicts the best-hidden state sequence, the Viterbi algorithm is used to identify and match the names of people and places.
10. In the above steps, the Chinese sentences are segmented into Chinese words, and the result is **R**=**w**_1_**w**_2_ ⋯ **w**_**M**_,  (1 ≤ **M** ≤ **N**).
11. Adjust word segmentation based on word segmentation and concatenation rules.

Based on the Chinese-Braille word corpus, the Chinese word segmentation is adjusted according to the Braille Word Segmentation and Concatenation Rules. The length of the word segmentation should be moderate, not too long, or too short. If it is too long and lacks a gap, the touch will easily cause fatigue and affect the effect of “touching and reading;” if it is too short, it is inconvenient to quickly form a concept and affect the speed of “touching and reading.” Because of this, the Braille Word Segmentation and Concatenation Rules has been developed in Braille, which is related to and different from Chinese word partitioning. For example, in the word “蒸馒头(steam steamed buns),” the Chinese segmentation is “蒸/馒头(steam/steamed buns),” and the word in Braille is a monosyllabic verb modifying a two-syllable noun, which needs to be written together. Therefore, Braille word segmentation is more coarse-grained than Chinese word segmentation.

Using the Chinese-Braille vocabulary corpus, a Braille word segmentation dictionary is established, and a unigram language model Braille word segmentation dictionary is obtained. Assume that **L**=**e**_1_**e**_2_ ⋯ **e**_**r**_,  (1 ≤ **r** ≤ **N**) is a Braille word segmentation dictionary, and the maximum matching algorithm is used to segment the Chinese sentence C. Because there is no binary language model, its ambiguity is difficult to eliminate.

Using the 2-gram model for Chinese word segmentation, the result is **R**=**w**_1_**w**_2_ ⋯ **w**_**M**_,  (1 ≤ **M** ≤ **N**), and **w**_**i**_ and **e**_**i**_ are a word of Chinese word segmentation and Braille word segmentation, respectively. Analysis and experiments show that *R* is fine-grained and disambiguates, while *L* is coarse-grained but difficult to disambiguate. The two results need to be fused so that the final result *R'* is coarse-grained and disambiguated.

**Definition 1 .**

Concatenated word. Given **w**_**i**,**i**+**k**_=**w**_**i**_**w**_**i**+1_ ⋯ **w**_**i**+**k**_, the segmentation results in a Chinese sentence **R**=**w**_1_**w**_2_ ⋯ **w**_**m**_,  (1 ≤ **m** ≤ **N**), and the Braille segmentation results **L**=**e**_1_**e**_2_ ⋯ **e**_**r**_,  (1 ≤ **r** ≤ **N**); if **w**_**i**,**i**+**k**_=**e**_**j**_, then **e**_**j**_ is called the concatenated word.

*Idea.* By default, the word segmentation is selected from *R* and placed in *R'.* When there combines *R* in *L*, the word segmentation of *L* is placed in *R'* (Algorithm 1).

#### 3.1.4. Chinese to Pinyin

As shown in Figure 1, the previously divided sentences are converted into Pinyin sequences. There are also relatively mature algorithms to realize the conversion of Chinese characters to Pinyin, but the biggest difficulty lies in polyphonic characters. Theoretically, if the pronunciation of the word is unique, it can be directly converted; if the number of pronunciations of the word is or greater than 2, the pronunciation of the word must be determined by the context.

Based on the Chinese sentence segmentation of the HanLP dictionary and the Chinese-Braille dictionary, the Chinese-Braille word corpus was generated. The Chinese-Braille word corpus used the probability to select the Pinyin sequence of words in the Chinese-Braille word corpus as the Pinyin sequence containing polyphonic words. A unigram language model is used for polysyllabic words in Braille dictionaries to reduce the problem of polysyllabic words.

The Pinyin sequence after Chinese word segmentation is still different from the Braille Pinyin sequence. The difference is not in Pinyin but in word segmentation (space position).

#### 3.1.5. Pinyin to Braille Pinyin Sequence

In order to convert Pinyin strings into Braille strings, based on the Chinese-Braille word corpus, a Pinyin-Braille syllable mapping table was established, and the Braille strings were obtained by searching the mapping table and replacing syllables one by one.

#### 3.1.6. Braille Pinyin Sequence to UTF-8

By searching the Chinese Common Braille Pinyin sequence of the Chinese-Braille word corpus, the initials, finals, and Braille ASCII codes are outputted in UTF-8 format after identifying the mapping relationship one by one.

### 3.2. Braille-Chinese Translation

When translating Braille into Chinese, there are mainly four categories including Chinese characters, English letters, numbers, and punctuation marks that need to be translated. There is a one-to-one mapping relationship between the Chinese Common Braille and English letters, numbers, and punctuation marks. If the Braille is accurate and there is no ambiguity, it can be directly converted, and the basic implementation is error-free, which will not be discussed here.

**Figure 4:**
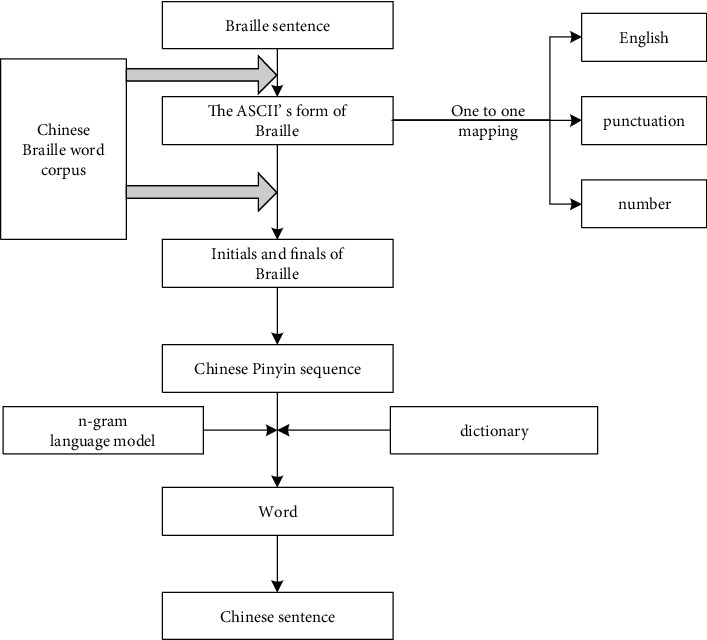
The translation process of Chinese common Braille-Chinese.

As shown in Figure 4, Braille to Chinese translation is the core. In the process of translation, Chinese Pinyin is used as the medium, and the difficulty lies in the homophones. The main process is listed below:

#### 3.2.1. Braille Recognition and Classification

For the input Braille (UTF-8) sentence, we get the corresponding UTF-8 code of each cell of Braille. We then handle punctuation that cut the Braille sequences into Braille sentences.

#### 3.2.2. Braille-Chinese's Pinyin Sequence

When converting Braille to the corresponding Chinese Common Braille Pinyin (initials and finals), the Braille characters have a strict one-to-one correspondence with initials and finals, making the accuracy of this step 100%. We then scan from the beginning to the end of the sentence according to the Chinese-Braille word corpus. The corresponding Chinese Pinyin can be obtained by looking up the Chinese Common Braille Pinyin, and the omitted tones can be supplemented (u2v, Pinyin, initials and finals, and other fields in Table 1).

Exceptions are as follows: (1) when “
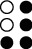
” appears alone, if the previous cell of Braille is not an initial, then it is a number symbol, and the latter cells are converted to numbers until the empty cell is found; (2) if it does not conform to the arrangement of initials, finals, and tones, and it is not a single syllable, it will be converted according to the English alphabet until an empty cell is found (ended with a concatenated word segmentation).

### 3.3. Pinyin-word Conversion

Suppose the Chinese Pinyin sequence **S**=**c**_1_**c**_2_ ⋯ **c**_**N**_， **c**_**i**_ is the Chinese Pinyin sequence of Braille word segmentation and concatenation. Spaces are used to separate **c**_**i**_**c**_**j**_.

Based on the Chinese-Braille vocabulary corpus, the maximum matching algorithm is used to convert the Pinyin sequence into Chinese words.

### 3.4. The Optimal Solution of Words to Form Sentences

When the Pinyin sequence is converted into Chinese words, there is a problem with polyphonic words. As shown in Table 4, after the previous processing, the Pinyin sequence bu4shi2 can be obtained, which can match “不时 (from time to time)” and “不识 (unknown),” in the Chinese-Braille vocabulary corpus. *L*(*S*) is a candidate sentence.

Using the 2-gram language model and HanLP's binary language model dictionary, the probability of occurrence of the entire word string is calculated. We then take the one with the highest probability as the result of sentence *R*.

(10) $$\begin{array}{c} R=\underset{l\in L\left(S\right)}{\max}P\left(l\right)=\underset{l\in L\left(S\right)}{\max}P\left(c_{1}\right)\prod_{k=2}^{N}P\left(c_{k}|c_{1},c_{2},\ldots ,c_{k-1}\right). \end{array}$$

Finally, the normative spaces of the word segmentation in *c*_*i*_*c*_*j*_ are deleted and are taken as the output.

## 4. Improve Braille Word Segmentation and Concatenation Rules

This section will present the experiment in the translation between Chinese and Braille and the fusion of Braille Word Segmentation and Concatenation Rules for the improvement of mutual translation. However, the word segmentation and concatenation rules of Braille are not yet mature [26], while the newly promulgated Chinese Common Braille is still being promoted; therefore, there is a lack of a real Braille corpus. We, therefore, proposed a scheme for improving Braille Word Segmentation and Concatenation Rules through artificial Chinese-Braille vocabulary corpus experiments [27].

The Braille Word Segmentation and Concatenation Rules are still immature. On the basis of the corpus, by improving the Braille word segmentation algorithm, a common unregistered word segmentation can be realized, to experimentally improve the Braille Word Segmentation and Concatenation Rules. Words that do not appear in the Braille dictionary still have some statistical information. Mikolov et al. proposed a method for word segmentation of English phrases [28]. Braille sentence expressions also use spaces (blank cells) for word segmentation. The method of extracting English phrases (similar to unregistered words) can be used to improve Braille Word Segmentation and Concatenation Rules.

Given two Braille words, if there exists a situation where the number of consecutive occurrences of *w*_*i*_, *w*_*j*_ count(*w*_*i*_, *w*_*j*_) is greater than the number of independent occurrences of *w*_*i*_*w*_*j*_ count(*w*_*i*_)  or count(*w*_*j*_); then, the two Braille words *w*_*i*_ and *w*_*j*_ are considered to be one Braille word; that is, the Braille words need to be linked together instead of being segmented. A threshold function can be defined accordingly:

(11) $$\begin{array}{c} f\left(w_{i},w_{j}\right)=\frac{\text{count}\left(w_{i},w_{j}\right)}{\text{count}\left(w_{i}\right)+\text{count}\left(w_{j}\right)}. \end{array}$$

Then, when *f* ≥ *x*, two consecutive Braille words *w*_*i*_ and  *w*_*j*_ will be merged into a new Braille segmentation *w*_*i*_*w*_*j*_, and the value of *x* needs to be set through experiments. The word vector is trained through statistical information such as the number of occurrences of Braille words and the number of simultaneous occurrences between words, to further improve the word segmentation effect and improve the Braille Word Segmentation and Concatenation Rules.

Suppose that *X*_*i*_ represents the number of all Braille word segmentations that appear in the context of the Braille word *w*_*i*_; *X*_*ij*_ represents the number of times the word *w*_*j*_ appears in the context of the word *w*_*i*_. Then,

(12) $$\begin{array}{c} X_{i}=\sum_{k}X_{ik}. \end{array}$$

The frequency of Braille segmentation *w*_*j*_ in the context of *w*_*i*_ is

(13) $$\begin{array}{c} P_{ij}=\frac{X_{ij}}{X_{i}}. \end{array}$$

We calculate the statistics of the cooccurrence times between Braille words and words in the Braille corpus, and the matrix of Braille word segmentation vector matrix is *A*. Then,

(14) $$\begin{array}{c} A\in R^{\left|V\right|\times d}, \end{array}$$

where |*V*| represents the number of Braille words, and *d* represents the dimension of the Braille word vector.

The above-mentioned Braille word vectors are large in scale, and a relatively simple model can be tested first as a training model, such as the GloVe model. The objective function trained with the GloVe model as the Braille word segmentation vector is

(15) $$\begin{array}{c} J\left(A\right)=\sum_{i,j}{\left(A_{i}^{T}A_{j}-\text{log}X_{ij}\right)}^{2}. \end{array}$$

To remove the low-frequency terms, the above objective function is improved, and the weight terms are added *f*(*X*_*ij*_).

(16) $$\begin{array}{c} f\left(X_{ij}\right)=\left\{\begin{array}{cc} {\left(\frac{X_{ij}}{X_{\max}}\right)}^{\alpha }, & X_{ij}<X_{\max},\\ 1, & \text{other.} \end{array}\right. \end{array}$$

After simplification, the objective function of word vector training is

(17) $$\begin{array}{c} J\left(A\right)=\sum_{i,j}f\left(X_{ij}\right){\left(A_{i}^{T}A_{j}-\log\;X_{ij}\right)}^{2}. \end{array}$$

The above Braille word segmentation training results can effectively improve the Braille Word Segmentation and Concatenation Rules.

Assume that Braille segmentation *w*_*i*_ (or synonym or congener of *w*_*i*_) and Braille segmentation *w*_*j*_ (or synonym or congener of *w*_*j*_) occur at high frequency (*w*_*i*_, *w*_*j*_), then the Braille segmentation *w*_*i*_ and the Braille segmentation *w*_*j*_ should be a new Braille word. If they do not exist in the corpus, they should be added to the Braille corpus.

**Assumption 1 .**

Braille segmentation *w*_*i*_ and *w*_*j*_ can form new words *w*_*i*_*w*_*j*_ or *w*_*j*_*w*_*i*_; then, the cosine value of the included angle between the word vectors *A*_*i*_ and *A*_*j*_ corresponding to the Braille segmentation *w*_*i*_ and the Braille segmentation *w*_*j*_ will be close to 1.

If the value obtained by calculation is greater than a certain threshold *λ* (specified via experiments), it is considered that the Braille segmentation *A*_*j*−1_ and the Braille segmentation *A*_*j*_ form a new Braille segmentation *A*_*j*−1_*A*_*j*_. The word segmentation vector is

(18) $$\begin{array}{c} A_{j-1}A_{j}=A_{j-1:j}=\frac{A_{j-1}+A_{j}}{\left|A_{j-1}+A_{j}\right|}. \end{array}$$

The above is to use the existing annotated Braille corpus for training and an experiment to improve the Braille Word Segmentation and Concatenation Rules. For Chinese word segmentation, this method simply uses statistical information and does not use the grammatical and semantic information of words.

## 5. Results

### 5.1. Examples of Translation

**Figure 5:**
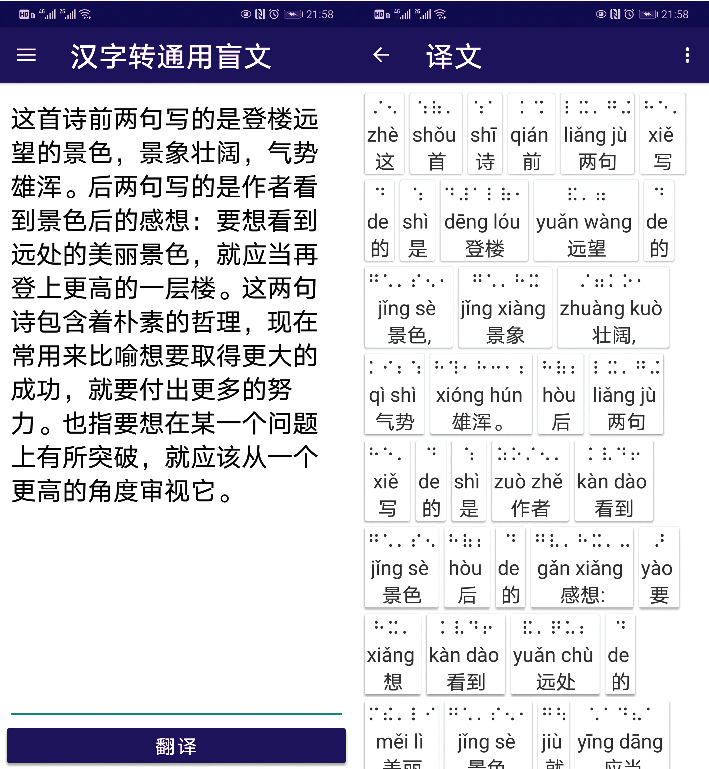
Examples of Chinese-Chinese Common Braille translation.

We tested the effect of translation between Chinese and Chinese Common Braille. The test set is the extracted sentences from books such as “Chinese Classics Reading (Large Character, Braille Edition)” published by China Braille Publishing House and the literary work “Looking Back-Fragments in Memory.” The translation program is shown in Figure 5, and the comparison between the translation result and the human translation is shown in Table 5.

### 5.2. Evaluation of Chinese-Braille Translation

Published by China Braille Publishing House, “Chinese Classics Reading (Large Characters, Braille Edition)” is a Chinese-Chinese Common Braille book, which belongs to the category of literature, with a small number of proper nouns, such as names of people and places. The Braille in the book is the Chinese Common Braille and is manually translated by Braille experts.

The BLEU (Bilingual Evaluation Understudy) evaluation index can be used to calculate the effect of machine translation [29].

(19) $$\begin{array}{c} \text{BLEU}=BP\cdot \;\exp\left(\sum_{n=1}^{N}w_{n}\log\;P_{n}\right), \end{array}$$

where

(20) $$\begin{array}{c} BP=\left\{\begin{array}{cc} 1, & \text{if }c>r,\\ e^{\left(1-r\right)/c}, & \text{if }c\leq r, \end{array}\right. \end{array}$$

where *c* is the number of Braille cells of the machine-translated Braille sentence, and *r* is the number of Braille cells of the Braille sentence translated by a Braille expert.

As can be seen from the translation results, an n-gram is fine-grained, and Braille Word Segmentation and Concatenation Rules are coarse-grained. All those that need to be concatenated have not been concatenated, and there are more blank cells, so *c* ≥ *r*. Then, *BP* *=* 1.

We extracted 1604 pairs of sentences (16995 Chinese characters and 40778 Braille) from the book using machine translation and compared the translation results. It can be seen from Table 6 that the BLEU increased by 12.19 and 4.65 after using the Chinese-Braille vocabulary corpus to establish a unigram language model Braille word segmentation and concatenation dictionary, and integrating the dictionary into n-gram, after incorporating the Braille characteristics of the word segmentation and concatenation rules. The host language of Braille is Chinese, so there are no grammatical problems in the translation process but mainly the problem of word segmentation and polyphonic words, so the BLEU value is relatively high. In Chinese-Braille translation, polyphonic words have little effect on the results, so the results are better.

### 5.3. Braille Word Segmentation Evaluation

**Figure 6:**
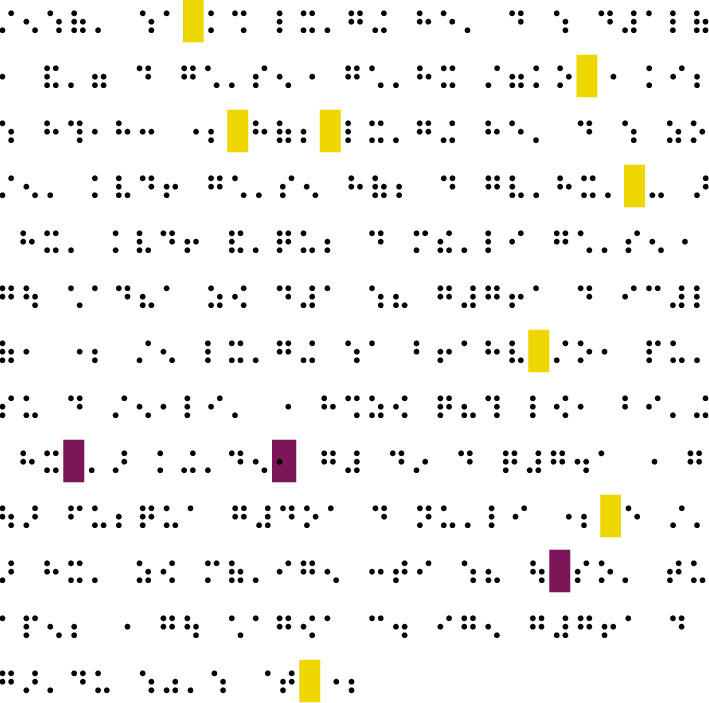
Error analysis of Chinese-Chinese Common Braille translation.

As can be seen from the translation results of Chinese to Braille, there are more mistakes in word segmentation; that is, the words that should be concatenated are not concatenated. As shown in Figure 6, the yellow (light color when printing in black and white) in the figure is the redundant word segmentation for machine translation (should be concatenated here), and purple (dark color when printing in black and white) is the word that should be segmented, or the tones of the words should be marked in machine translation. Therefore, the quality of the translation mainly relies on the word segmentation of Braille. The evaluation metrics of Chinese word segmentation can be used to evaluate Braille word segmentation.

The evaluation metrics are accuracy, recall, precision, and F1. It is assumed that the correct number of words (the number of Braille cells) converted into Braille after segmentation processing is TP; TP + FP is the total number of Braille cells converted into Chinese after segmentation; TP + FN is the total number of Braille cells after expert manual Braille word segmentation [25]. Spaces (blank cells) are counted in all calculations. When calculating the accuracy rate, we aligned the Braille characters of the machine translation (*T* text) with the human translation (*H* text), the total number of Braille characters after alignment is *N*, and the inconsistency between *T* and *H* is called the substitution error (SN). It is called an insertion error (IN) when *T* has a word that H has not while it is an omission error (ON) when *H* has a word that *T* has not.

The Braille to Chinese translation process is similar to the above, and the calculation formula is (21)–(24).

Accuracy *A*:

(21) $$\begin{array}{c} A=\frac{N-SN-\text{IN}-\text{ON}}{N}. \end{array}$$

Precision *P*:

(22) $$\begin{array}{c} P=\frac{TP}{TP+FP}. \end{array}$$

Recall rate *R*:

(23) $$\begin{array}{c} R=\frac{TP}{TP+FN}. \end{array}$$

F1:

(24) $$\begin{array}{c} F1=\frac{2\times P\times R}{P+R}. \end{array}$$

We then conducted experiments based on the electronic copy of the book “Chinese Classics Reading (Large Character, Braille Edition).”

We then tested the effect of Chinese to Braille machine translation. Due to the limited size of the corpus, the training corpus is comprised of the HanLP dictionary and the Chinese-Braille word segmentation and concatenation dictionary generated from the Chinese-Braille word corpus. The corpus of Chinese Braille covers the fields of education and literature. The test set comes from “Chinese Classics Reading (Large Character · Braille Version).”

It should be noted that the content of the test set is consistent with the field of the Chinese-Braille word corpus, but the Chinese-Braille word corpus does not have the training data from “Chinese Classics Reading (Large Character Braille Version).” As shown in Table 7, the accuracy of Chinese to Braille translation is 95.01%, which is 4.99% higher than the traditional method; the F1 value is 97.41%, which is 3.93% higher than the traditional method. In the process of translating from Braille to Chinese, the accuracy rate is 90.15%, which is 0.74% higher than the traditional method; the F1 value is 91.45%, which is 0.75% higher than the traditional method.

## 6. Discussion

This study proposes a Chinese-Braille translation method that integrates word segmentation and concatenation rules. Firstly, the n-gram language model is used to perform Chinese word segmentation, and then, the Chinese-Braille vocabulary corpus is used to train and generate a Braille word segmentation and concatenation dictionary of a unigram language model to adjust the Braille word segmentation and improve the translation results between Chinese and Chinese Common Braille.

Experiments show that in the field of educational literature, the accuracy of translation from Chinese to Chinese Common Braille has reached 95.01%, and the accuracy of Chinese Common Braille to Chinese translation has reached 90.15%.

This research also has some limitations. One is that the effect of Braille to Chinese translation is not significantly improved. The main reason is that the resource-restrained corpus makes the model training insufficient, and the situation of typos is serious, especially the single-character polyphonic words (such as he, she, it) that have serious errors. The translation performance failed to improve effectively even after the utilization of the Chinese-Braille word corpus. In future work, we will build a corpus of Braille sentences and use deep learning methods for training, which may result in better performance.

The Braille Word Segmentation and Concatenation Rules is not perfect. At present, word segmentation and concatenation mainly rely on manual annotation by Braille experts. If a large-scale Braille corpus can be established, it is possible to improve the word segmentation and concatenation rules of the Chinese Common Braille through experiments.

## Figures and Tables

**Algorithm 1:**
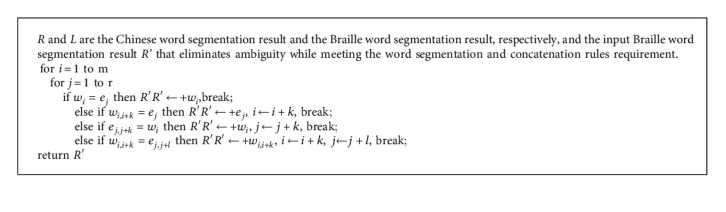
Chinese word segmentation results adjusted to word segmentation and concatenation rules.

**Table 1:** Field of Chinese-Braille word corpus (parts).

Corpus field	Explanation	Example
BSCR	Braille Word Segmentation and Concatenation Rules	CD
u2v	Chinese Pinyin, *u* to *v*	geng4/jia1
Pinyin	The Pinyin's form of Chinese Common Braille. The abbreviation rules of tone	geng/jia1
Initials and finals	Initials and finals of Braille	geng/jia1
ASCII's of Braille	The ASCII's form of Braille	G#G$A
Common Braille	The Unicode's form of Chinese Common Braille	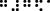

**Table 2:** A list of word segmentation and concatenation marks (parts).

Index	Classification	Symbol	Word frequency	Sample
1	Noun	N	6897	人、品德、蓝色、国家、思路、心胸、机器、阿胶、思想家、阿司匹林
	Noun of location	Nf	277	上、下、左、右、东、西、里面、南、北、之中
	Place NOUN	Nd	2587	日本国、韩国、北京、北京市、夏威夷、唐古拉山、三峡、加州、华北
	Personal noun	Nr	1840	唐太宗、马克思、豆豆、鲁迅、黑旋风、卡尔·马龙、小布什
	Other proper nouns	Nz	1639	北京大学、英语、抗日战争、肯德基、左传、诺贝尔奖、和谐号、长城饭店、海豹突击队、开国大典

2	Verb	V	15196	走、爱、调查、同意、喜爱、包含、跨
	Directional verb	V		来、回、上去、下来、出去、进来、出来

3	Adjective	A	3863	美丽、丑陋、雪白、公共、皑皑、金灿灿

4	Numeral	M	7041	一、一百五十一、第一、一百零八、百分之十

5	Classifier	Q	82	个、沓、千克、架次、册、吨、朵、光年、赫兹

6	Adverb	D	4669	很、必、已经、处处、单独、倍加、必定、不妨
	Special adverb		-	不_Dbu_

7	Pronoun	R	1859	我、我们、她、这个、什么、怎样、这么、谁、哪里

**Table 3:** Statistical information of Braille word segmentation and concatenation corpus.

The sum of word frequencies in the corpus	229551
Number of words	31708
Number of Common Braille with Pinyin	1477
The sum of Common Braille word frequencies in the same Pinyin	52762
The sum of the highest word frequencies of Common Braille words in the same Pinyin	46235
The number of Chinese characters in Common Braille with the same Pinyin	3491

**Table 4:** Fragments of Braille word segmentation and concatenation dictionary.

Pinyin	Chinese words	Braille Word Segmentation and Concatenation Rules	ASCII code of Braille	Word frequency
bu4/shi	不是	DbuV/JC	BU:	276
bu4/shi	不事	DbuV	BU:	1
bu4/shi	不适	CD	BU:	4
bu4/shi	不释	I2	BU:	2
bu4/shi2	不时	CD	BU:1	23
bu4/shi2	不识	DbuV	BU:1	2
bu4/shi2/pu	不识谱	DbuVN	BU : 1PUʹ	1

**Table 5:** Comparison of Chinese-Chinese Common Braille translation.

Chinese sentence	这使我们都很惊奇。
Braille expert translation	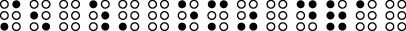
	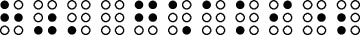

Translation methods of this paper	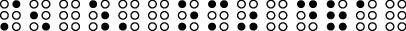
	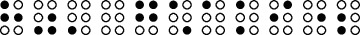

**Table 6:** Comparison of BLEU between Chinese-Chinese Common Braille translation.

Method	BLEU%	
	Chinese-Braille translation	Braille-Chinese translation
n-gram	70.97	61.57
n-gram + segmentation concatenation rules	**83.16**	**66.22**

**Table 7:** Comparison of effect between Chinese-Chinese Common Braille Translation.

	Segmentation method	*A*%	*P*%	*R*%	F1%
Chinese-Braille translation	n-gram	90.02	93.95	93.02	93.48
	n-gram + segmentation concatenation rules	**95.01**	96.46	98.38	**97.41**

Braille-Chinese translation	n-gram	89.41	89.92	91.49	90.70
	n-gram + segmentation concatenation rules	**90.15**	90.84	92.07	**91.45**

## Data Availability

The Chinese Common Braille corpus is provided by Zhong Jing-Hua's team at Beijing Union University. The data has not been fully disclosed.
